# Multivariate Correlation Analysis of the Electroconductive Textiles Obtained Using Functionalization by Radio-Frequency Oxygen Plasma Treatments

**DOI:** 10.3390/ma14195609

**Published:** 2021-09-27

**Authors:** Raluca Maria Aileni, Laura Chiriac, Doina Toma, Irina Sandulache

**Affiliations:** 1 Department of Research in Textile Materials Engineering and Processes, National Research and Development Institute for Textiles and Leather, 030508 Bucharest, Romania; laura.chiriac@incdtp.ro (L.C.); doina.toma@incdtp.ro (D.T.); 2 Department of Material Research and Investigation, National Research and Development Institute for Textiles and Leather, 030508 Bucharest, Romania; irina.sandulache@incdtp.ro

**Keywords:** plasma, coating, conductive, thin film, textile, multivariate correlation analysis

## Abstract

This paper presents a study concerning the preliminary treatments in radiofrequency (RF)oxygen (O_2_) plasma used to obtain a hydrophilic effect on raw cotton fabrics followed by electroconductive thin film deposition to obtain electroconductive textile surfaces. In addition, this study presents a multivariate correlation analysis of experimental parameters. The treatment using RF plasma O_2_ aimed to increase the hydrophilic character of the raw fabric and adherence of paste-based polymeric on polyvinyl alcohol (PVA) matrix and nickel (Ni), silver (Ag) or copper (Cu) microparticles. The purpose of the research was to develop electroconductive textiles for flexible electrodes, smart materials using a clean technology such as radiofrequency (RF) plasma O_2_ to obtain a hydrophilic surface with zero wastewater and reduced chemicals and carbon footprint. To achieve the foreseen results, we used advanced functionalization technologies such as RF plasma O_2_, followed by scraping a thin film of conductive paste-based Ni, Ag or Cu microparticles, and multivariate correlation methods to observe the dependence between parameters involved (dependent and independent variables). Overall, the fabrics treated in plasma with O_2_ using a kHz or MHz generator and power 100–200 W present an excellent hydrophilic character obtained in 3 min. After RF O_2_ plasma functionalization, a thin film based on polymeric matrix PVA and Ni microparticles have been deposited on the fabric surface to obtain electroconductive materials.

## 1. Introduction

Plasma treatment was successfully used in numerous applications for textile cleaning [1,2] or hydrophobization. Primarily, plasma activation using work gas oxygen was used for polyester to increase the wettability [3,4,5] or improve the polypyrrole adhesion [6]. Moreover, for cotton fabrics [7,8,9,10], RF plasma oxygen was used to generate the surface activation for preliminary surface preparation to be treated with ZnO nanoparticles [11,12] or titanium dioxide [13,14]. In addition, several types of research present the use of plasma O_2_ treatment to improve the metal absorption [15,16] or to generate an antibacterial [17,18,19] or antimicrobial activity [20] on cotton fabric modified by low-temperature plasma. Numerous investigations use plasma to create electroconductive surfaces [21] using silver nanoparticles and polypyrrole [22] or copper [23]. Mainly plasma was preferred because it is a friendly environmental technology having zero waste and a reduced carbon footprint. In addition, at the laboratory or industrial stage, surface modification can be performed using low-pressure plasma and atmospheric-pressure plasma [24]. Some scientific papers present some tests using the gaseous mixtures (Ar/O_2_ and He/O_2_) plasma activation of polyester (PES) or poly (ethylene terephthalate) fabrics to improve the hydrophilic and aging effects [25,26,27]. The material’s surface becomes hydrophilic using plasma O_2_ activation due to the formation of polar groups (C=O, O-C=O) on the surface [27,28].

In some cases, the discharge diagnostics are carried out, such as the applied voltage shape or waveform [29,30,31] dependence on the barrier discharge. In our experiment, the dependencies current and voltage on time were not investigated.

The main goal of this study was to develop textile materials with electroconductive properties to be used as flexible electrodes by applying a preliminary treatment based on RF plasma O_2_ technology on the raw fabrics with a fibrous composition of 100% cotton, followed by coating with a thin film based on polymeric matric polyvinyl alcohol (PVA) and nickel microparticles. In addition, a laboratory testing program has been elaborated to examine the electrical surface resistance; the physical-mechanical characteristics such as air permeability, vapor permeability, thickness and mass; and the influence of the RF plasma O_2_ finishing processes in comparison with classical finishing processes such as alkaline boiling-bleaching for obtaining a hydrophilic effect of the textile surface.

## 2. Materials and Methods

**Figure 1:**
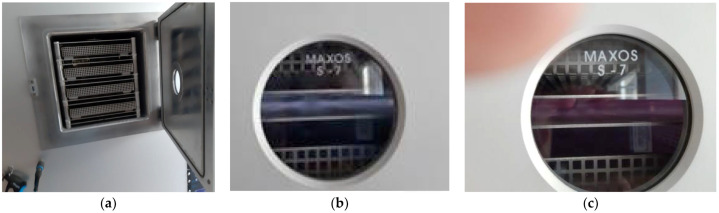
RF plasma O_2._ (**a**) vacuum chamber with cassettes; (**b**) RF plasma O_2_ using RF_1_ generator 13.56 MHz; (**c**) RF plasma O_2_ using RF_2_ generator 40 kHz.

We used the advanced functionalization technology RF plasma oxygen (Figure 1) to achieve the foreseen results and scrap conductive paste-based nickel on the textile surface. The experiments were performed using a low-pressure RF-based plasma system (CD400 PLC Europlasma, Oudenaarde, Belgium). The low-pressure plasma technique creates plasma using an RF generator, which utilizes the energy of an electrical field to dissociate a process gas under vacuum conditions. The parallel ground and positive electrodes are mounted in the vacuum chamber (Figure 1a), separated by ceramic spacers. The voltage supply of the system is connected to the electrodes on the backside of the system by RF-feedthrough. The plasma state is obtained by applying a high voltage (500–550 V) between the ground and RF electrode in the radiofrequency (RF) range. However, the voltage value cannot be set from the software interface menu. In the vacuum chamber, the textile materials are placed in cassettes (Figure 1a) made of aluminum and have 400 × 400 mm dimensions.

During the process, electrical energy from the RF generator is applied to these electrodes, which excites the oxygen (O_2_) present in the vacuum chamber. The dissociated gas with excited and unstable species modifies the fabric surface.

Mainly, a higher power creates higher activity of the excited species. In our experiments, we used the constants such as time 3 min and gas flow 200 sccm, varying only the RF power generator types (RF1 having 13.56 MHz frequency or RF2 having 40 kHz frequency), pressure, and power to observe the hydrophilic effects generated on the textile surfaces treated in RF plasma O_2_.

The textile and electrical properties such as wettability and electrical resistance were investigated using adequate devices such as the Video Camera Angle (VCA)-Optima (AST Products, Inc., Billerica, MA, USA) device and Surface Resistance Tester Warmbier METRISO B530 (Wolfgang Warmbier GmbH & Co., Hilzingen, Germany)—with concentric ring probes. To analyze the dependence between dependent and independent variables, correlation and statistical variation methods were used.

**Figure 2:**
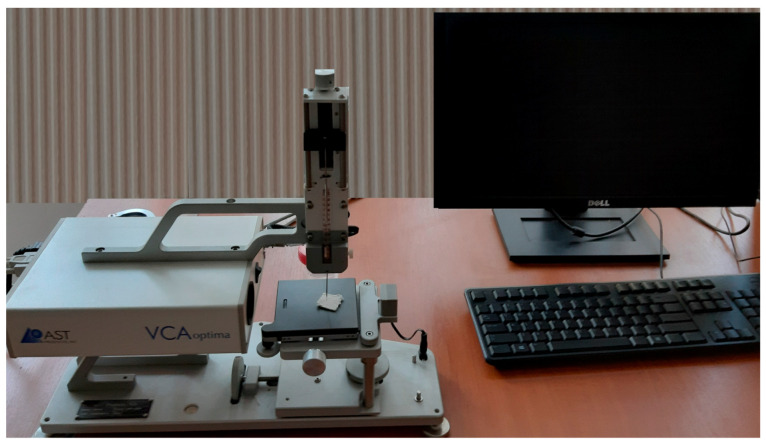
VCA Optima device for contact angle investigation (AST products).

In the experiment, 22 samples(INCDTP, Bucharest, Romania) (100% cotton and 100% polyamide fabrics) were treated in RF O_2_ plasma using an RF_1_ generator (Seren IPS Inc., Vineland, NJ, USA) in MHz or RF_2_ kHz followed by scraping with a paste-based polymeric PVA and distilled water (dH_2_O), organic solvents from Sigma Aldrich, Taufkirchen, Germany (Acetone, Ethanol, C_4_H_8_O_2_) and Ni microparticles (Sigma Aldrich, Taufkirchen, Germany); drying at room temperature and crosslinking for 3–5 min. From all samples, 8 samples were treated on RF O_2_ plasma using an RF_1_ generator in MHz, and an RF_2_ generator was used for 14 samples, followed by scraping with a paste, drying and crosslinking. All samples were electrically evaluated and were treated in plasma using the specific plasma process parameters (Table 1). For samples 1–22, the following parameters are used for multivariate analysis: surface resistance (Rs), mass (M), thickness (δ), vapor permeability (Pv) and air permeability (Pa) (Table 1). The samples used for RF plasma O_2_ functionalization and presented in Table 1 have been developed based on seven weaved structures from 100% cotton yarns such as plain weave 1/1 with cotton yarns Nm 20/2 on weft for samples 3, 11 and 19; plain weave 1/1 with cotton yarns Nm 20/3 on weft for samples 4, 12 and 20; twill 2/2 for samples 1, 9 and 17; twill 3/1 for samples 2, 10 and 18; panama for samples 5, 13 and 21; weft rib weave for samples 6, 14 and 22; and warp rib weave for samples 7 and 15. In addition, samples 5 and 16 are made of polyamide and weaved structure plain weave 1/1. Of samples 1–16 preliminary treated in RF plasma O_2_, samples 1–8 used RF_1_ generator and power 200 W, and samples 9–16 used RF plasma O_2_ with RF_2_ generator and 100 W power. However, the weaved structures do not influence the surface resistance. For all samples developed being obtained, the values of 10^3^ Ω for surface resistance mean that the conductivity is good, and the materials obtained are electroconductive. For samples 17–22, it can be observed that the surface resistance is between 10^12^–10^13^ Ω which means that samples 17–22 are electrical insulators. The samples presented in Table 1 have been developed based on seven weaved structures from 100% cotton yarns (plain weave 1/1 with yarns Nm20/2 (3, 11 and 19), plain weave 1/1 with cotton yarns Nm 20/3 (4, 12 and 20), twill 2/2 (1, 9 and 17), twill 3/1 (2, 10 and 18), panama (5, 13 and 21), weft rib weave (6, 14 and 22), and warp rib weave (7, 15)). The values for physico-mechanical and electrical tests performed before deposition of the conductive paste may be caused by different treatments performed in plasma by varying the RF generator, power and work gas pressure and the variation of thickness for the conductive layer deposited by scrapping method on the textile surface. Moreover, we observed, by measuring the contact angles using the VCA Optima device (AST Products, Inc., Billerica, MA, USA) (Figure 2) using a drop of 4 µL distilled water, that the samples treated using RF_1_ generator and power 200 W andRF_2_ generator and power 100 W present a pronounced hydrophilic character in comparison with the samples treated using the RF_2_ generator having a frequency of 40 kHz and power 50 W.

**Figure 3:**
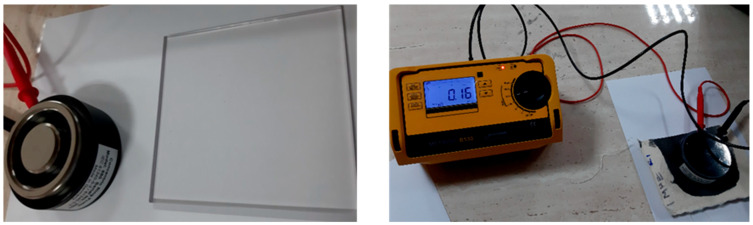
METRISO B530 Surface Resistance Tester and concentric ring probe 880.

The surface resistances were evaluated using two devices:

1. To measure the Rs[Ω] in Table 1 was used a portable Surface Resistance Meter (Quantachrome, Beijing, China) and the surface resistance (Rs [Ω]) was measured as the ratio between the continuous voltage applied between the two parallel electrodes on the surface of a test sample and the current between these electrodes, neglecting any polarization phenomena on the electrodes.
2. Surface resistance (noted with R_EDS_ [Ω]) was measured using a high precision device Warmbier METRISO B530 Surface Resistance Tester (Wolfgang Warmbier GmbH & Co., Hilzingen, Germany) based on concentric electrodes (Figure 3) and supply voltage:
  - 10 V for samples treated in RF plasma O_2_ using an RF_2_ generator 40 kHz and power of 100 W, respectively, an RF_1_ generator of 13.56 MHz frequency and power 200 W;
  - 100 V for samples treated in RF plasma O_2_ using an RF_2_ generator of 40 kHz frequency and power 50 W.

In the case of samples 1–16 (Table 1), preliminarily treated using RF plasma O_2_ using RF_1_ MHz generator and power 200 W or RF_2_ generator and 100 W power, the fabrics being conductive, the R_ESD_ data was captured for 10 V supply voltage. In the case of the samples 17–22 (Table 1), preliminarily treated using RF_2_ kHz generator and power 50 W, and the fabrics being electrical insulators, R_ESD_ data acquisition was possible only for a supply tension of 100 V.

Several raw fabrics presented in Table 2 were treated using the preliminary classical cleaning method alkaline boiling-bleaching (samples A1–A3) and alkaline boiling-bleaching followed by hydrophobization using a dispersion of fluorine compound NUVA TCC (CLARIANT AG, Muttenz, Switzerland) (samples A4–A6). After initial treatment on the fabrics, a thin-film based polymeric matrix PVA, organic solvents and silver (Ag), nickel (Ni), copper (Cu) has been deposited in A1–A6 (Table 2) by scraping followed by free drying at room temperature (18–20 °C) for 20–24 h and crosslinking at 150–160 °C for 3–5 min (Table 2). For these samples, the main physical-mechanical characteristics are presented in Table 2, i.e., mass (M), thickness (δ) and air permeability (Pa). In addition, for samples presented in Table 2, the electrical surface resistance (Rs_1_ [Ω] measured before crosslinking and Rs_2_ [Ω] measured after crosslinking) was measured. Samples A1–A6 have been developed based on 3 weaved structures from 100% cotton yarns: twill 2/2 for samples A1 and A4, twill 3/1 for samples A2 and A5, and plain weave 1/1 with cotton yarns Nm 20/2 on weft for samples A3 and A6.

The purpose of the experiments was to compare the classical alkaline boiling-bleaching followed by conductive paste scraping (samples A1–A3), the classical alkaline boiling-bleaching followed by hydrophobization using NUVA TTC and conductive paste scraping (samples A4–A6) and the conductive paste deposition after functionalization in RF plasma O_2_ to avoid classical cleaning treatments that harm air and water through intensive pollution and high chemical consumption. From Table 2, we can observe that the samples treated with conductive paste-based Ni and Ag are conductive because the surface resistance is 10^3^ Ω and are not dependent on the surface type (hydrophilic or hydrophobic). In the case of the samples A1 and A4 having initial surfaces that are hydrophilic and hydrophobic, respectively, and treated with a conductive paste-based Cu, we can observe that the surface resistances after crosslinking are 10^12^ Ω and 10^10^ Ω, respectively, which means that sample A1 has an insulator character, and sample A4 has an antistatic character.

## 3. Results

### 3.1. Characterization of the Samples Treated Using RF Plasma O_2_ and Conductive Paste

#### 3.1.1. Surface Morphology Using SEM

The surface morphology was obtained by scan electron microscopy Quanta 200 SEM (FEI, Cleveland, OH, USA) using 2000× magnification.

**Figure 4:**
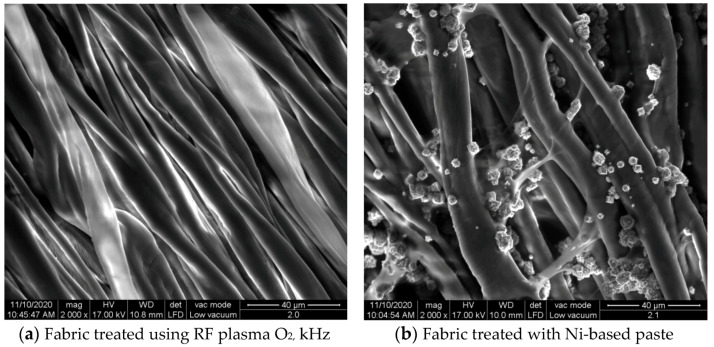
The sample was treated using RF_2_ plasma O_2_ using kHz generator and power 50 W. (**a**) represents the image of the sample treated using RF plasma O_2_ kHz and power 50 W, respective (**b**) represents the sample treated with Ni-based paste.

**Figure 5:**
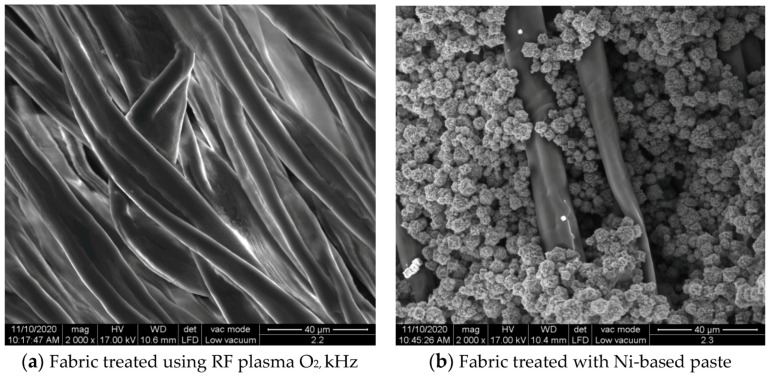
The sample was treated using RF_1_ plasma O_2_ using MHz generator and power 200 W. (**a**) represents the image of the sample treated using RF plasma O_2_ MHz and power 200 W, respective (**b**) represents the sample treated with Ni-based paste.

**Figure 6:**
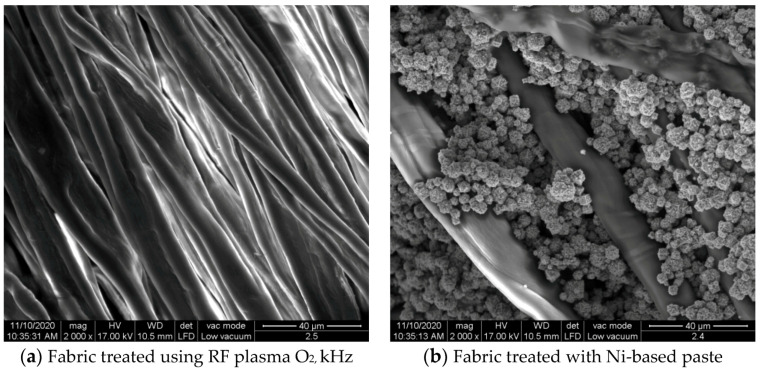
The sample was treated using RF_2_ plasma O_2_ using kHz generator and power 100 W. (**a**) represents the image of the sample treated using RF plasma O_2_ kHz and power 100 W, respective (**b**) represents the sample treated with Ni-based paste.

**Figure 7:**
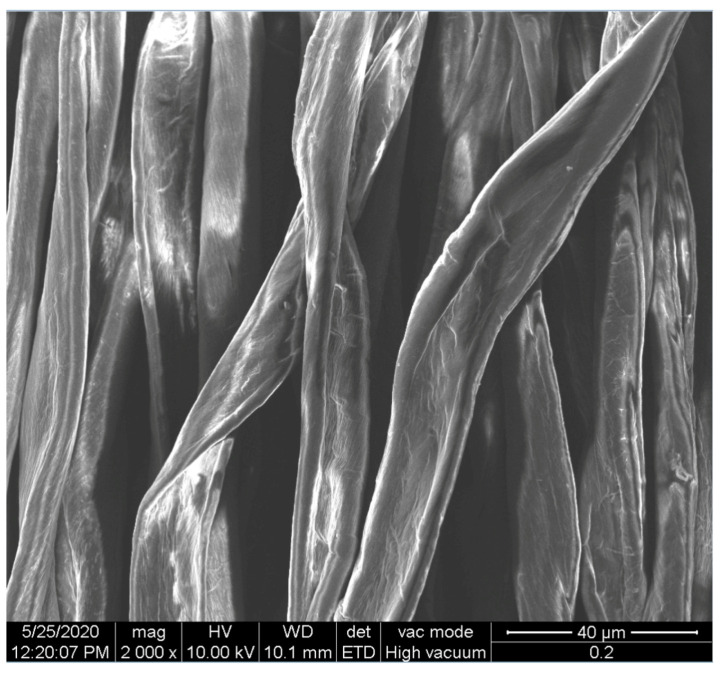
Raw fabric sample—structure twill 3/1 used to obtain samples 2, 10 and 18 from Table 1.

The surface morphologies of the fabrics treated using RF plasma O_2_ and samples coated with conductive paste-based polymeric matrix and Ni are presented in Figure 4, Figure 5 and Figure 6 (corresponding to the samples 18, 2 and 10 from Table 1). In Figure 5b and Figure 6b, a clusterization of the Ni microparticles is evident. The sample from Figure 5b was treated using RF plasma and MHz generator and exhibited a pronounced hydrophilic effect. However, we can observe in Figure 5b a massive clusterization in the case of the sample preliminarily treated using RF plasma O_2_ and RF_1_ MHz generator and power 200 W in comparison with the sample treated using RF_2_ kHz generator and power 50 W (Figure 4b). In addition, we can observe in Figure 6b and Figure 4b that Ni microparticles are more clusterized in the case of the fabric preliminarily treated with RF plasma O_2_ and generator RF_2_ and power 100 W (Figure 6b) in comparison with the sample treated using RF_2_ kHz generator and power 50 W (Figure 3b). Figure 7 is presented the raw fabric (100% cotton, twill 3/1 structure used initially for samples 18, 2 and 10 from Table 1) untreated in plasma and used to obtain samples 2, 10, 18 from Table 1. In comparison with samples presented in Figure 4a and Figure 6a (treated in RF plasma), we can observe that the surfaces of the samples treated in plasma look smoother and have a reduced surface roughness at the fiber surface level.

#### 3.1.2. Surface Topography Using Optical Microscopy

Table 3 presents the analysis of the topography of textile surfaces based on digital electron microscopy (60× magnification) for raw fabrics hydrophilized in RF plasma O_2_ using a 13.56 MHz RF_1_ generator and materials with polymeric deposits based on PVA matrix and nickel (Ni) microparticles. In Table 4 are presented the images, obtained through digital electron microscopy (60× magnification), of the samples obtained by classical technologies (boiling-bleaching (samples 1–3) and hydrophobization using NUVA TTC (samples 4–6)) and scraping of conductive pastes based on PVA matrix and Ni, Cu and Ag microparticles. For all samples, the scraping size is the same in all the samples and the photos in Table 3.

#### 3.1.3. Chemical Composition

The chemical composition was investigated using the ESD (Energy Dispersive Spectroscopy) analysis and elemental mapping overlay for samples 18, 2 and 10 (Table 1) performed using Energy Dispersive X-Ray Analysis (EDAX) (AMETEK, Inc., Montvale, NJ, USA) and the results are presented in Table 5. Using the smart phase mapping tool from EDAX the spectra and elemental and phase maps were collected automatically. These samples from Table 1 are noted here:

- Sample 18 from Table 1 is S1;
- Sample 2 from Table 1 is S2;
- Sample 10 from Table 1 is S3.

**Figure 8:**
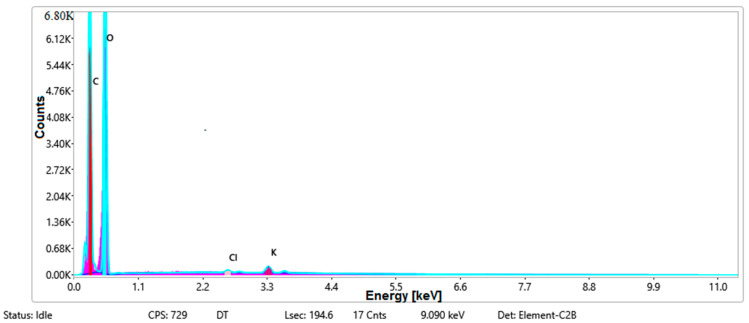
EDS spectra for sample S1 treated in RF plasma O_2_, using RF_2_ generator and power 50 W.

**Figure 9:**
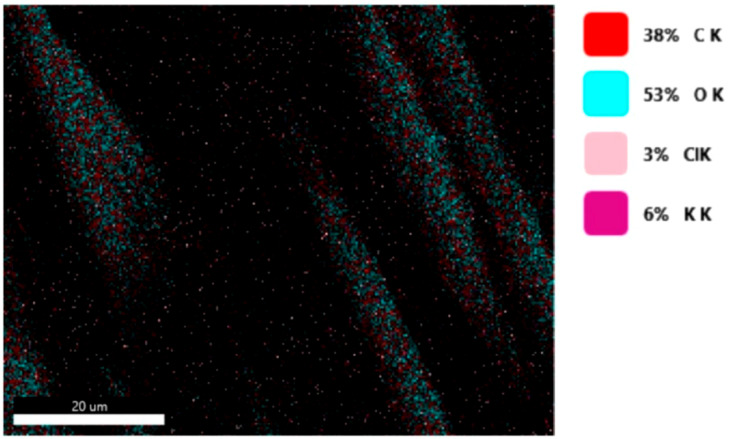
Smart elemental mapping overlay for sample S1 treated in RF plasma O_2_, using RF_2_ generator and power 50 W.

**Figure 10:**
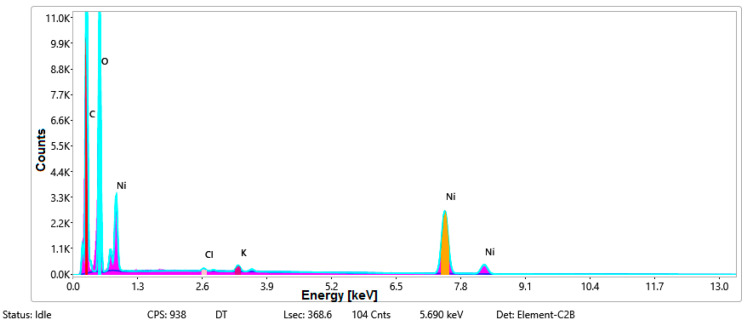
EDS spectra for sample S1 treated in RF plasma O_2_, using RF_2_ generator and power 50 W and Ni paste.

**Figure 11:**
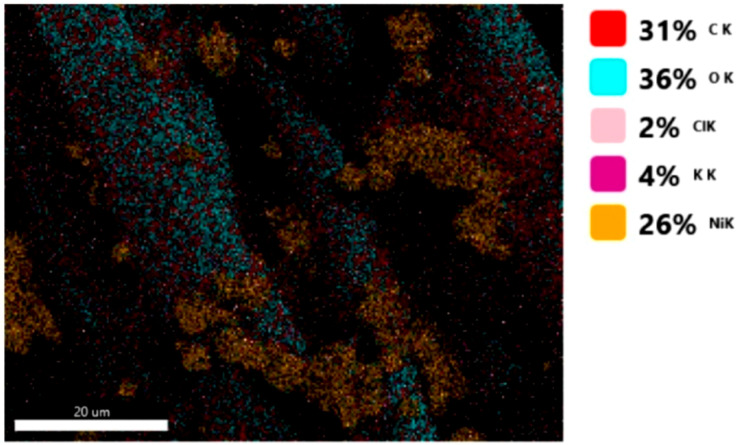
Smart elemental mapping overlay for sample S1 treated in RF plasma O_2_, using RF_2_ generator and power 50 W and Ni paste.

**Figure 12:**
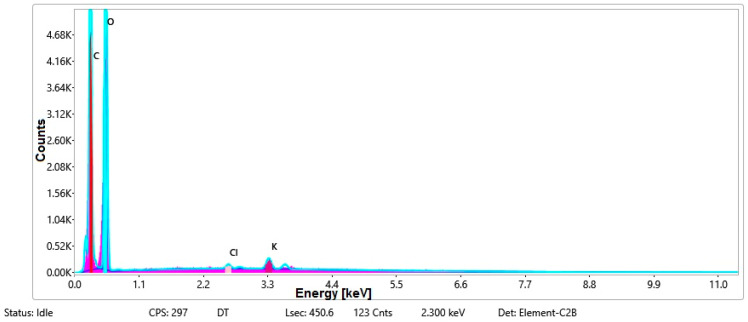
ESD spectra for sample S2 treated in RF plasma O_2_, using RF_1_ generator and power 200 W.

**Figure 13:**
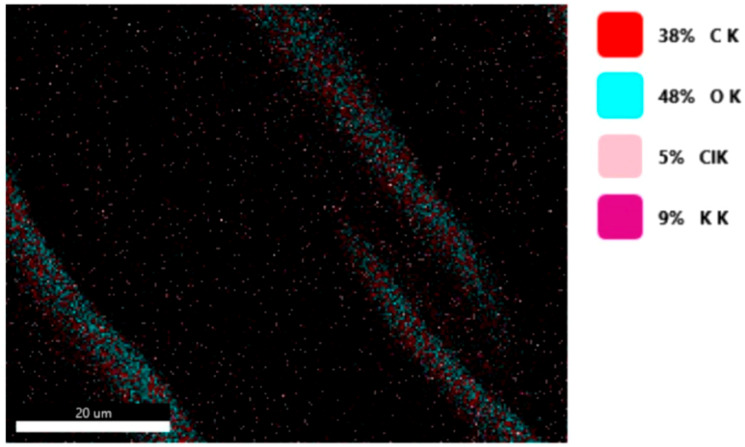
Smart elemental mapping overlay for sample S2 treated in RF plasma O_2_, using RF_1_ generator and power 200 W.

**Figure 14:**
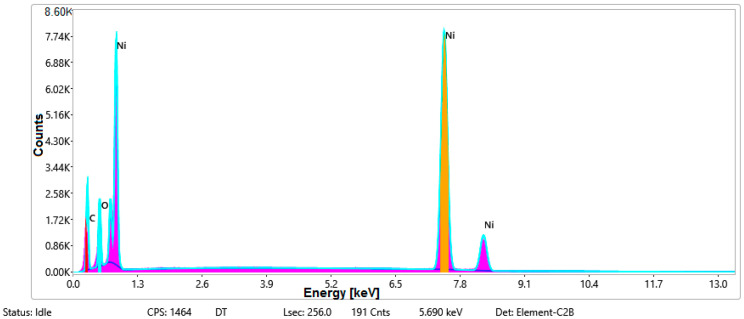
ESD spectra and elemental overlay for sample S2 treated in RF plasma O_2_, using RF_1_ generator and power 200 W and Ni paste.

**Figure 15:**
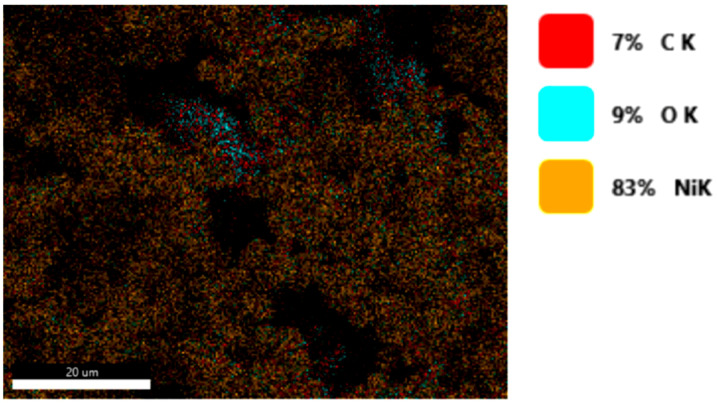
Smart elemental mapping overlay for sample S2 treated in RF plasma O_2_, using RF_1_ generator and power 200 W and Ni paste.

**Figure 16:**
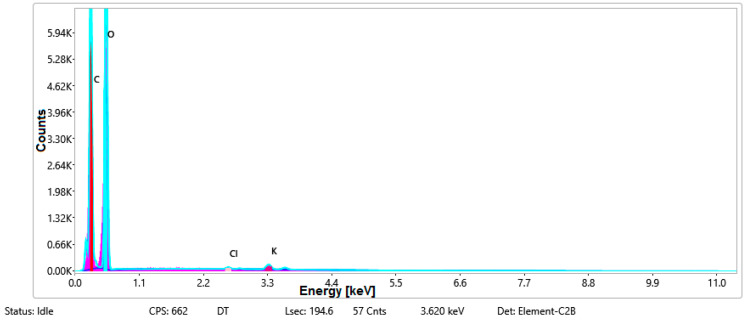
ESD spectra for sample S3 treated in RF plasma O_2_, using RF_2_ generator and power 100 W.

**Figure 17:**
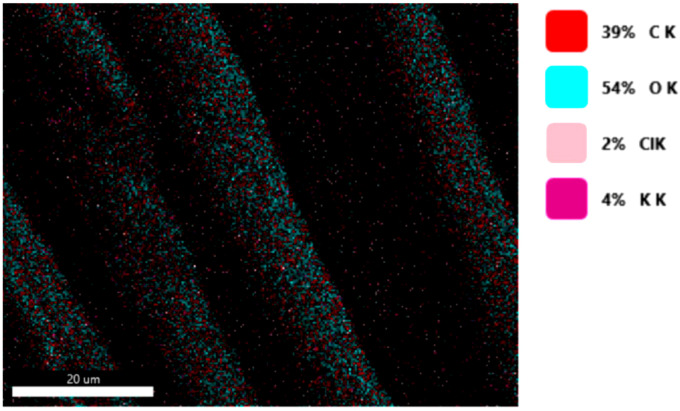
Smart elemental mapping overlay for sample S3 treated in RF plasma O_2_, using RF_2_ generator and power 100 W.

**Figure 18:**
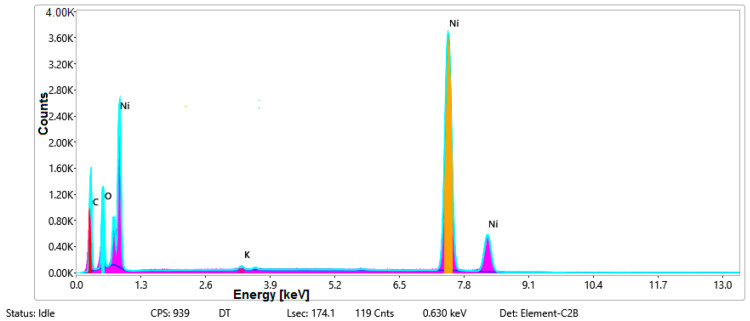
ESD spectra and elemental overlay for sample S3 treated in RF plasma O_2_, using RF_2_ generator and power 100 W and Ni paste.

**Figure 19:**
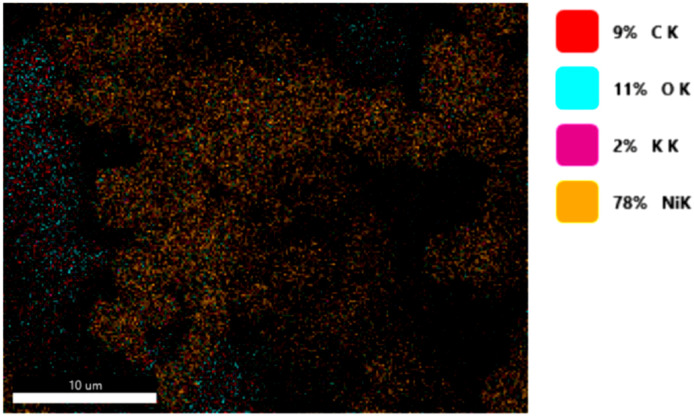
Smart elemental mapping overlay for sample S3 treated in RF plasma O_2_, using RF_2_ generator and power 100 W and Ni paste.

Figure 8, Figure 9, Figure 10 and Figure 11 present the ESD spectra (Figure 8 and Figure 10) and the element smart mapping (Figure 9 and Figure 11) for sample no. S1 treated in RF plasma using a kHz generator and power 50 W (Figure 8 and Figure 9) and then coated with conductive paste-based Ni microparticles (Figure 10 and Figure 11). Figure 12, Figure 13, Figure 14 and Figure 15 present the ESD spectra (Figure 12 and Figure 14) and the element smart mapping (Figure 13 and Figure 15) for sample S2 treated in RF plasma using RF_1_ generator and power 200 W (Figure 12 and Figure 13) and treated using RF plasma O_2_ followed by coating with Ni-based paste (Figure 14 and Figure 15). Figure 16, Figure 17, Figure 18 and Figure 19 present the ESD spectra (Figure 16 and Figure 18) and the element smart mapping (Figure 17 and Figure 19) for sample S3 treated in RF plasma using RF_2_ generator and power 100 W (Figure 16 and Figure 17) and treated using RF plasma O_2_ followed by coating with Ni-based paste (Figure 18 and Figure 19). The prominent peaks of C and O (Figure 8, Figure 10, Figure 12, Figure 14, Figure 16 and Figure 10) are because cotton fibers contain 94% cellulose ((C6H10O5)n), and other substances such as protein, pectin substance, oil, fat, wax, ash, organic acids [32]. In addition, the cotton bolls contain an approximate percent of 4% potassium (K) which is necessary to the growth and development of cotton [33,34,35]. The energy peak corresponding to the element Cl in ESD spectra (Figure 8, Figure 10, Figure 12 and Figure 16) on the ESD spectra mean that Cl and K element is present on the fabric analyzed and can have the provenience from the substances used for warps preparation (gluing the warps for preparation of the yarns to be used on warp direction) before weaving). Figure 10, Figure 14 and Figure 18 show that Ni peaks for samples treated with conductive paste-based Ni microparticles can be observed. By comparison, it can be observed that the Ni peaks are more pronounced in Figure 14 (corresponding to the sample treated in RF plasma O_2_ using RF_1_ generator and 200 W power) than Figure 18 (corresponding to the sample treated in RF plasma O_2_ using RF_2_ generator and 100 W power).

From Figure 11, it is evident that the adherence of the paste and distribution of the 26% Ni is lower on the sample S1 treated in RF plasma using RF_2_ and 50 W power in comparison with the samples treated in RF plasma using RF_1_ generator and power 200 W (Figure 16 and Figure 17) and RF_2_ generator and power 100 W (Figure 18 and Figure 19). In Figure 15 and Figure 19, it can be observed that Ni has a good mapping and tends to create appropriate clusters on the surface of the sample S2 (83%) and sample S3 (78%).

## 4. Discussion

The experiments in RF plasma O_2_ were prepared using a variation in power (200 W, 100 W and 50 W), in frequency (using two different RF generators having the frequencies 13.56 MHz (RF_1_) and 40 kHz (RF_1_)).

The samples treated in RF plasma O_2_ using the generator RF_1_ and power 200 W become hydrophilic. In addition, the samples treated in RF plasma O_2_ using the generator RF_2_ and power 100 W become hydrophilic.

On the other hand, the samples treated in RF plasma O_2_ using the generator RF_2_ and power 50 W continued to be hydrophobic.

The correlation between surface resistance after free drying or crosslinking (dependent variable) and other independent variables (Pa, Pv, δ and M) was investigated by analyzing the correlation coefficient Pearson (1) between R_ESD_ and M, Pa, Pv and δ. The correlation coefficients were calculated for all samples preliminarily treated using RF plasma O_2_ with RF_1_ generator and power 200 W, RF_2_ and power 100 W, and RF_2_ and power 50 W:

(1) $$r_{xy}=\frac{\frac{1}{n}\sum \left(x-\bar{x}\right)\left(y-\bar{y}\right)}{s_{x}s_{y}}$$

where:

*x*, *y* represents the individual values of the variables *x* and *y*;
$\bar{x},\bar{y}$ represents the arithmetic mean of all the values of *x*, *y*;
*s_x_*, *s_y_* represents the standard deviation of all values *x* and *y*.

**Figure 20:**
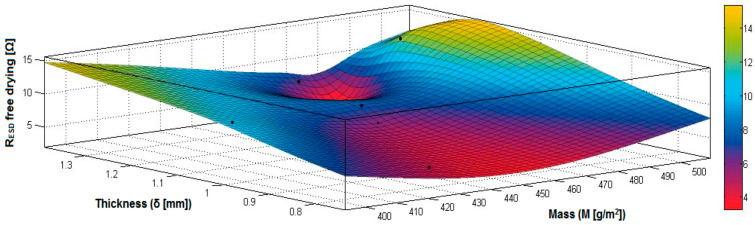
3D representation R_ESD free drying_ = f(M, δ) samples preliminarily treated in RF plasma O_2_ using RF_1_ generator and power 200 W.

**Figure 21:**
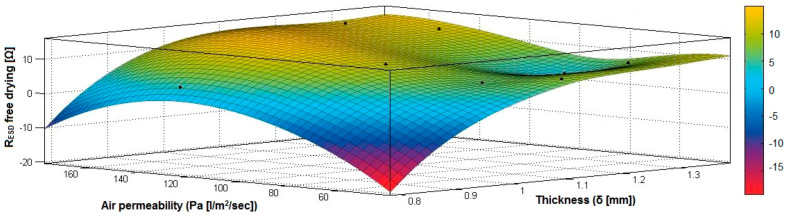
3D representation R_ESD free drying_ = f(Pa, δ) samples preliminarily treated in RF plasma O_2_ using RF_1_ generator and power 200 W.

**Figure 22:**
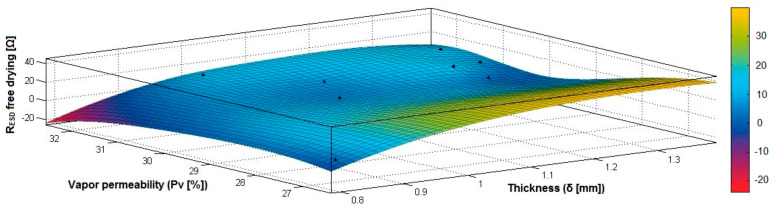
3D representation R_ESD free drying_ = f(Pv, δ) for samples preliminarily treated in RF plasma O_2_ using RF_1_ generator and power 200 W.

**Figure 23:**
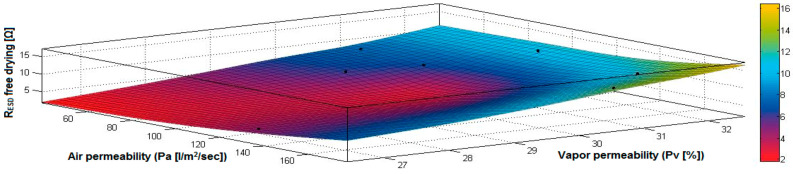
3D representation R_ESD free drying_ = f(Pa, Pv) for samples preliminarily treated in RF plasma O_2_ using RF_1_ generator and power 200 W.

**Figure 24:**
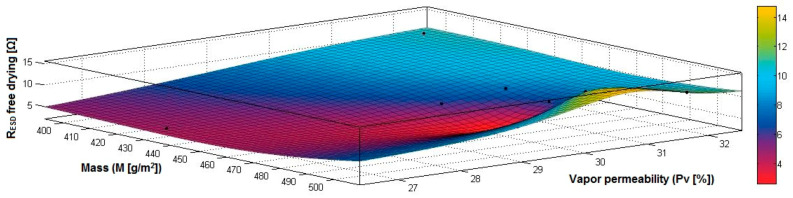
3D representation R_ESD free drying_ = f(M, Pv) for samples preliminarily treated in RF plasma O_2_ using RF_1_ generator and power 200 W.

**Figure 25:**
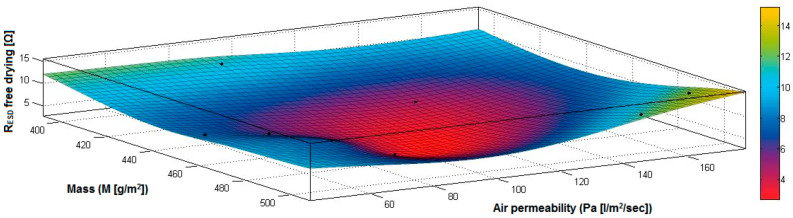
3D representation R_ESD free drying_ = f(M, Pa) for samples preliminarily treated in RF plasma O_2_ using RF_1_ generator and power 200 W.

In the case of the fabrics treated in RF plasma using RF_1_ and power 200 W, Figure 20, Figure 21, Figure 22, Figure 23, Figure 24 and Figure 25 present the 3D representation of the surface resistance (R_ESD free drying_), after free drying at room temperature (18–20 °C) for 20–24 h, depending on the independent variables (mass and thickness (Figure 20), air permeability and thickness (Figure 21), vapor permeability and thickness (Figure 22), air permeability and vapor permeability (Figure 23), mass and vapor permeability (Figure 24) and mass and air permeability (Figure 25) to observe the variation of the R_ESD free drying_ in correlation with these independent variables. To appreciate the inverse or direct correlation between the dependent variable R_ESD free drying_ and independent variables (mass, thickness, vapor permeability and air permeability), the correlation coefficients between the R_ESD free drying_ and mass (2), air permeability (3), vapor permeability (4) and thickness (5) were calculated.

Analyzing the values of the correlation coefficients between surface resistance (R_ESD_) after free drying and M, Pa, Pv and δ ($r_{R_{ESD},\;M}$ = 0.2209 (2), $r_{R_{ESD},\;Pa}$ = 0.5307 (3), $r_{R_{ESD},\;Pv}$ = 0.5993 (4) and $r_{R_{ESD},\;\delta }$ = 0.2921 (5)), it can be observed that the correlation coefficients are positive. Between the surface resistance (R_ESD_) and air permeability (Pa) and vapor permeability (Pv), it is a direct correlation, and this indicates that a textile fabric having higher values for Pa and Pv surface will generate the increase of surface resistance value. Moreover, between R_ESD_ and thickness and mass, it is an insignificant dependence.

Figure 20 presents the 3D representation of the multiple correlations between R_ESD free drying_ and (M, δ) and the black dots represent the appropriate points for R_ESD free drying_ depending on mass (M) and thickness (δ).

Figure 21 presents the 3D representation of the multiple correlations between R_ESD free drying_ and (Pa, δ) and the black dots represent the fitting points for R_ESD free drying_ depending on air permeability (Pa) and thickness (δ).

Figure 22 presents the 3D representation of the multiple correlations between R_ESD free drying_ and (Pv, δ) and the black dots represent the fitting points for R_ESD free drying_ depending on vapor permeability (Pv) and thickness (δ).

Figure 23 presents the 3D representation of the multiple correlations between R_ESD free drying_ and (Pa, Pv) and the black dots represent the fitting points for R_ESD free drying_ depending on air permeability (Pa) and vapor permeability (Pv).

Figure 24 presents the 3D representation of the multiple correlations between R_ESD free drying_ and (M, Pv) and the black dots represent the fitting points for R_ESD free drying_ depending on mass (M) and vapor permeability (Pv).

Figure 25 presents the 3D representation of the multiple correlations between R_ESD free drying_ and (M, Pa) and the black dots represent the fitting points for R_ESD free drying_ depending on mass (M) and air permeability (Pa).

(2) $$r_{R_{ESD},\;M}=\left|\begin{array}{cc} 1.0000 & 0.2209\\ 0.2209 & 1.0000 \end{array}\right|\Leftrightarrow r12_{R_{ESD},M}=r21_{R_{ESD},M}=0.2209$$

(3) $$r_{R_{ESD},\;Pa}=\left|\begin{array}{cc} 1.0000 & 0.5307\\ 0.5307 & 1.0000 \end{array}\right|\Leftrightarrow r12_{R_{ESD},Pa}=r21_{R_{ESD},Pa}=0.5307$$

(4) $$r_{R_{ESD},\;Pv}=\left|\begin{array}{cc} 1.0000 & 0.5993\\ 0.5993 & 1.0000 \end{array}\right|\Leftrightarrow r12_{R_{ESD},Pv}=r21_{R_{ESD},Pv}=0.5993$$

(5) $$r_{R_{ESD},\;\delta }=\left|\begin{array}{cc} 1.0000 & 0.2921\\ 0.2921 & 1.0000 \end{array}\right|\Leftrightarrow r12_{R_{ESD},\delta }=r21_{R_{ESD},\delta }=0.2921$$

**Figure 26:**
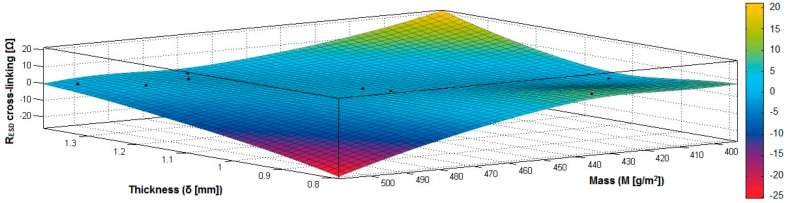
3D representation R_ESD after crosslinking_ = f(M, δ) samples preliminarily treated in RF plasma O_2_ using RF_1_ generator and power 200 W.

**Figure 27:**
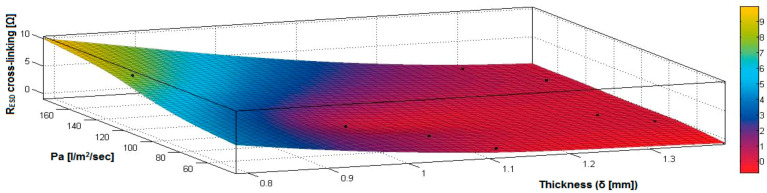
3D representation R_ESD after crosslinking_ = f(Pa, δ) samples preliminarily treated in RF plasma O_2_ using RF_1_ generator and power 200 W.

**Figure 28:**
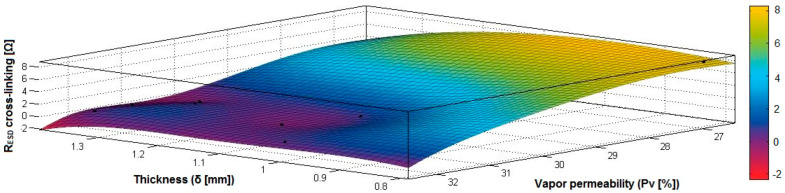
3D representation R_ESD after crosslinking_ = f(Pv, δ) samples preliminarily treated in RF plasma O_2_ using RF_1_ generator and power 200 W.

**Figure 29:**
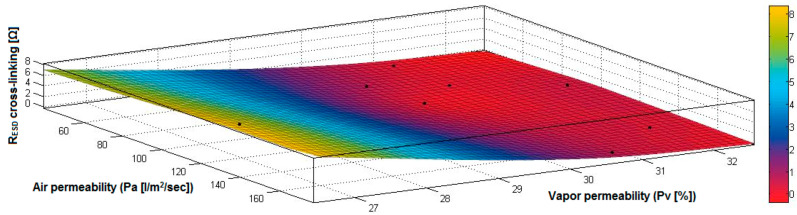
3D representation R_ESD after crosslinking_ = f(Pa, Pv) samples preliminarily treated in RF plasma O_2_ using RF_1_ generator and power 200 W.

**Figure 30:**
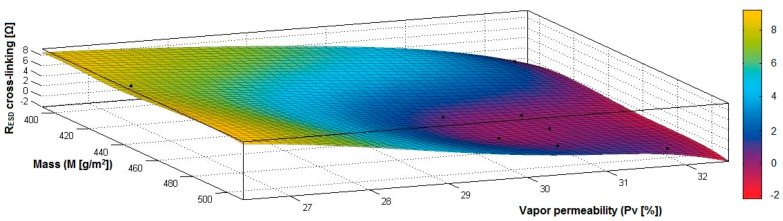
3D representation R_ESD after crosslinking_ = f(M, Pv) samples preliminarily treated in RF plasma O_2_ using RF_1_ generator and power 200 W.

**Figure 31:**
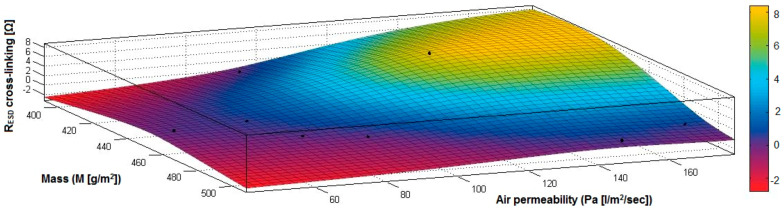
3D representation R_ESD after crosslinking_ = f(M, Pa) samples preliminarily treated in RF plasma O_2_ using RF_1_ generator and power 200 W.

In the case of the fabrics treated in RF plasma using RF_1_ and power 200 W, Figure 26, Figure 27, Figure 28, Figure 29, Figure 30 and Figure 31 present the 3D representation of the surface resistance (R_ESD after crosslinking_), after crosslinking, depending on the independent variables (mass and thickness (Figure 26), air permeability and thickness (Figure 27), vapor permeability and thickness (Figure 28), air permeability and vapor permeability (Figure 29), mass and vapor permeability (Figure 30), mass and air permeability (Figure 31)) to observe the variation of the dependent variable R_ESD after crosslinking_ in correlation with these independent variables. To evaluate the inverse or direct correlation between the dependent variable R_ESD after crosslinking_ and independent variables (mass, thickness, vapor permeability and air permeability), the correlation coefficients between the R_ESD crosslinking_ and mass (6), air permeability (7), vapor permeability (8), and thickness (9) were calculated.

By analysis of the values for correlation coefficients between surface resistance (R_ESD_) after crosslinking and M, Pa, Pv and δ ($r_{R_{ESD},\;M}$ = −0.3497 (6), $r_{R_{ESD},\;Pa}$= 0.2917 (7), $r_{R_{ESD},\;Pv}$ = −0.9086 (8), $r_{R_{ESD},\;\delta }$ = −0.7475 (9)), it can be observed that between the surface resistance (R_ESD_) and vapor permeability (Pv), thickness (δ) and mass (M), the correlation coefficients are negative, and the inverse correlation indicates that in the case of a textile fabric coated with a conductive paste-based Ni, the increase of the values for δ, Pv and M will generate a reduction of the R_ESD_ value. However, between R_ESD_ and Pa, the correlation coefficient is moderately positive, which means a direct insignificant dependence between these variables.

Figure 26 presents the 3D representation of the multiple correlations between R_ESD after crosslinking_ and (M, δ) and the black dots represent the fitting points for R_ESD after crosslinking_ depending on mass (M) and thickness (δ).

Figure 27 presents the 3D representation of the multiple correlations between R_ESD after crosslinking_ and (Pa, δ) and the black dots represent the fitting points for R_ESD after crosslinking_ depending on air permeability (Pa) and thickness (δ).

Figure 28 presents the 3D representation of the multiple correlations between R_ESD after crosslinking_ and (Pv, δ) and the black dots represent the fitting points for R_ESD after crosslinking_ depending on vapor permeability (Pa) and thickness (δ).

Figure 29 presents the 3D representation of the multiple correlations between R_ESD after crosslinking_ and (Pa, Pv) and the black dots represent the fitting points for R_ESD after crosslinking_ depending on air permeability (Pa) and vapor permeability (Pv).

Figure 30 presents the 3D representation of the multiple correlations between R_ESD after crosslinking_ and (M, Pv) and the black dots represent the fitting points for R_ESD after crosslinking_ depending on mass (M) and vapor permeability (Pv).

Figure 31 presents the 3D representation of the multiple correlations between R_ESD after crosslinking_ and (M, Pa) and the black dots represent the fitting points for R_ESD after crosslinking_ depending on mass (M) and air permeability (Pa).

(6) $$r_{R_{ESD},\;M}=\left|\begin{array}{cc} 1.0000 & -0.3497\\ -0.3497 & 1.0000 \end{array}\right|\Leftrightarrow r12_{R_{ESD},M}=r21_{R_{ESD},M}=-0.3497$$

(7) $$r_{R_{ESD},\;Pa}=\left|\begin{array}{cc} 1.0000 & 0.2917\\ 0.2917 & 1.0000 \end{array}\right|\Leftrightarrow r12_{R_{ESD},Pa}=r21_{R_{ESD},Pa}=0.2917$$

(8) $$r_{R_{ESD},\;Pv}=\left|\begin{array}{cc} 1.0000 & -0.9086\\ -0.9086 & 1.0000 \end{array}\right|\Leftrightarrow r12_{R_{ESD},Pv}=r21_{R_{ESD},Pv}=-0.9086$$

(9) $$r_{R_{ESD},\;\delta }=\left|\begin{array}{cc} 1.0000 & -7475\\ -0.7475 & 1.0000 \end{array}\right|\Leftrightarrow r12_{R_{ESD},\delta }=r21_{R_{ESD},\delta }=-0.7475$$

**Figure 32:**
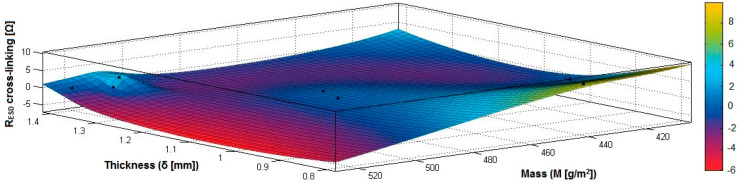
3D representation R_ESD after crosslinking_ = f(M, δ) samples preliminarily treated in RF plasma O_2_ using RF_2_ generator and power 100 W.

**Figure 33:**
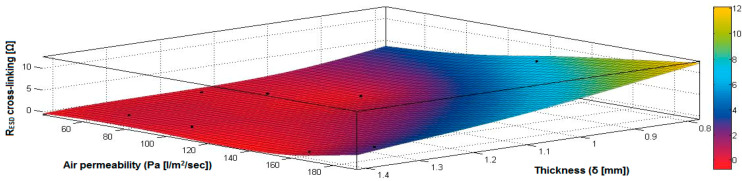
3D representation R_ESD after crosslinking_ = f(Pa, δ) samples preliminarily treated in RF plasma O_2_ using RF_2_ generator and power 100 W.

**Figure 34:**
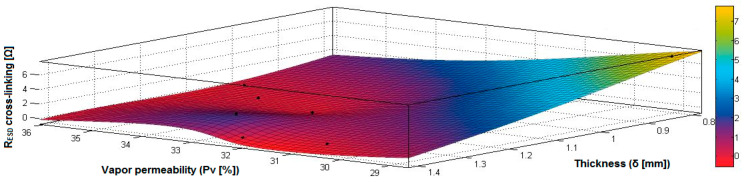
3D representation R_ESD after crosslinking_ = f(Pv, δ) samples preliminarily treated in RF plasma O_2_ using RF_2_ generator and power 100 W.

**Figure 35:**
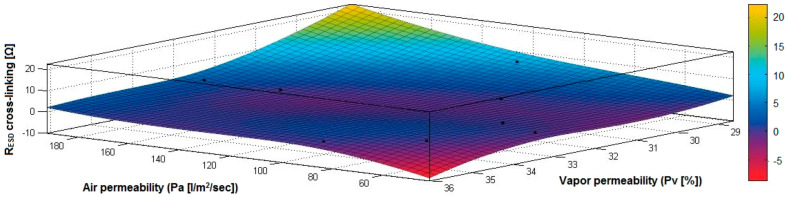
3D representation R_ESD after crosslinking_ = f(Pa, Pv) for samples preliminarily treated in RF plasma O_2_ using RF_2_ generator and power 100 W.

**Figure 36:**
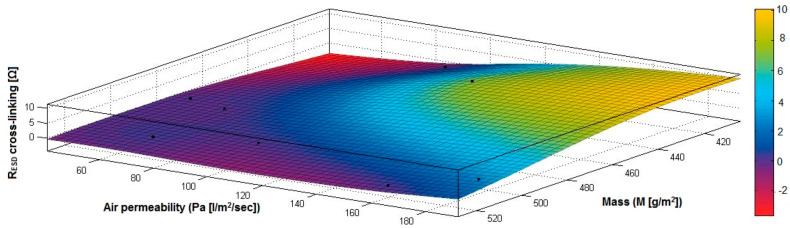
3D representation R_ESD after crosslinking_ = f(M, Pa) for samples preliminarily treated in RF plasma O_2_ using RF_2_ generator and power 100 W.

**Figure 37:**
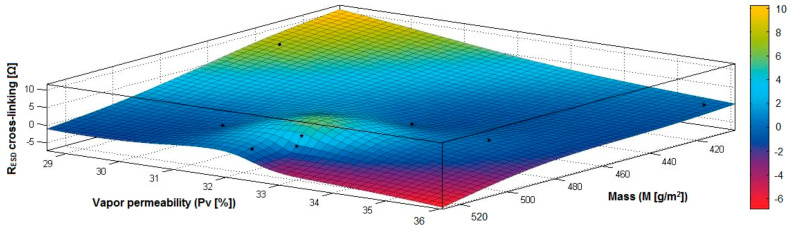
3D representation R_ESD_ = f(M, Pv) for samples preliminarily treated in RF plasma O_2_ using RF_2_ generator and power 100 W.

In the case of the fabrics treated in RF plasma using RF_2_ and power 100 W, Figure 32, Figure 33, Figure 34, Figure 35, Figure 36 and Figure 37 present the 3D representation of the surface resistance (R_ESD after crosslinking_), after crosslinking, depending on the independent variables (mass and thickness (Figure 32), air permeability and thickness (Figure 33), vapor permeability and thickness (Figure 34), air permeability and vapor permeability (Figure 35), mass and vapor permeability (Figure 36) and mass and air permeability (Figure 37) to observe the variation of the R_ESD after crosslinking_ in correlation with the independent variables (mass, thickness, vapor permeability and air permeability).

In the case of the samples preliminarily treated using RF plasma O_2_ with RF_2_ generator and power 100 W, the correlation coefficients (10)–(13) have been calculated:

(10) $$r_{R_{ESD},\;M}=\left|\begin{array}{cc} 1.0000 & -0.3093\\ -0.3093 & 1.0000 \end{array}\right|\Leftrightarrow r12_{R_{ESD},M}=r21_{R_{ESD},M}=-0.3093$$

(11) $$r_{R_{ESD},\;Pa}=\left|\begin{array}{cc} 1.0000 & 0.3934\\ 0.3934 & 1.0000 \end{array}\right|\Leftrightarrow r12_{R_{ESD},Pa}=r21_{R_{ESD},Pa}=0.3934$$

(12) $$r_{R_{ESD},\;Pv}=\left|\begin{array}{cc} 1.0000 & -0.6898\\ -0.6898 & 1.0000 \end{array}\right|\Leftrightarrow r12_{R_{ESD},Pv}=r21_{R_{ESD},Pv}=-0.6898$$

(13) $$r_{R_{ESD},\;\delta }=\left|\begin{array}{cc} 1.0000 & -0.5845\\ -0.5845 & 1.0000 \end{array}\right|\Leftrightarrow r12_{R_{ESD},\delta }=r21_{R_{ESD},\delta }=-0.5845$$

Analyzing the values of the correlation coefficient $r_{R_{ESD},\;Pa}$(11), which is positive, it can be observed that between the surface resistance (R_ESD_) and air permeability (Pa), there is a direct correlation, and these variables are in a direct proportionality relationship, and this indicates that the increase of the Pa value generates the increase of the R_ESD_ and reduction of the electrical conductivity. In addition, we can observe that between R_ESD_ and M, Pv and δ, the correlation coefficients are negative. The inverse correlation indicates that increasing the value of the parameters M, Pv and δ will reduce surface resistance and increase the conductivity.

Figure 32 presents the 3D representation of the multiple correlations between R_ESD after crosslinking_ and (M, δ) and the black dots represent the fitting points for R_ESD after crosslinking_ depending on mass (M) and thickness (δ).

Figure 33 presents the 3D representation of the multiple correlations between R_ESD after crosslinking_ and (Pa,δ) and the black dots represent the fitting points for R_ESD after crosslinking_ depending on air permeability (Pa) and thickness (δ).

Figure 34 presents the 3D representation of the multiple correlations between R_ESD after crosslinking_ and (Pv,δ) and the black dots represent the fitting points for R_ESD after crosslinking_ depending on vapor permeability (Pv) and thickness (δ).

Figure 35 presents the 3D representation of the multiple correlations between R_ESD after crosslinking_ and (Pa, Pv) and the black dots represent the fitting points for R_ESD after crosslinking_ depending on air permeability (Pa) and vapor permeability (Pv).

Figure 36 presents the 3D representation of the multiple correlations between R_ESD after crosslinking_ and (M, Pa) and the black dots represent the fitting points for R_ESD after crosslinking_ depending on mass (M) and air permeability (Pa).

In Figure 37 presents the 3D representation of the multiple correlations between R_ESD after crosslinking_ and (M, Pv) and the black dots represent the fitting points for R_ESD after crosslinking_ depending on mass (M) and vapor permeability (Pv).

**Figure 38:**
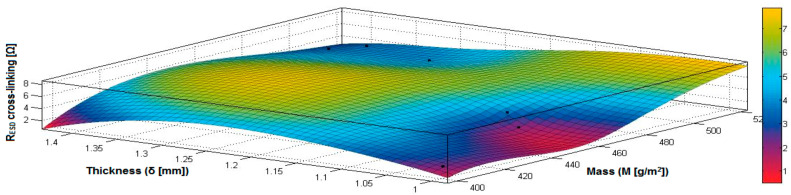
3D representation R_ESD after crosslinking_ = f(M, δ) samples preliminarily treated in RF plasma O_2_ using RF_2_ generator and power 50 W.

**Figure 39:**
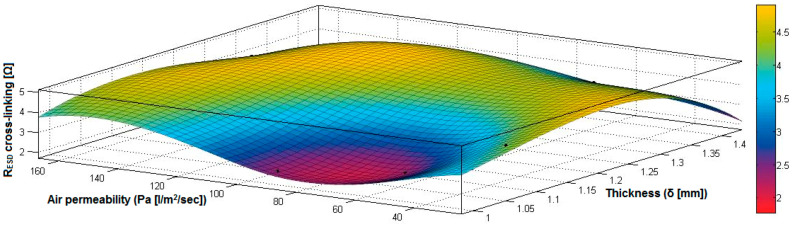
3D representation R_ESD after crosslinking_ = f(Pa, δ) samples preliminarily treated in RF plasma O_2_ using RF_2_ generator and power 50 W.

**Figure 40:**
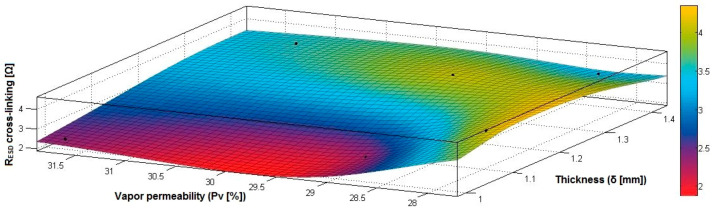
3D representation R_ESD after crosslinking_ = f(Pv, δ) samples preliminarily treated in RF plasma O_2_ using RF_2_ generator and power 50 W.

**Figure 41:**
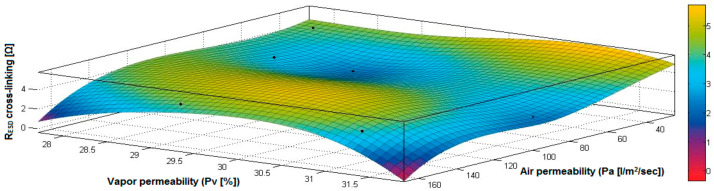
3D representation R_ESD after crosslinking_ = f(Pa, Pv) samples preliminarily treated in RF plasma O_2_ using RF_2_ generator and power 50 W.

**Figure 42:**
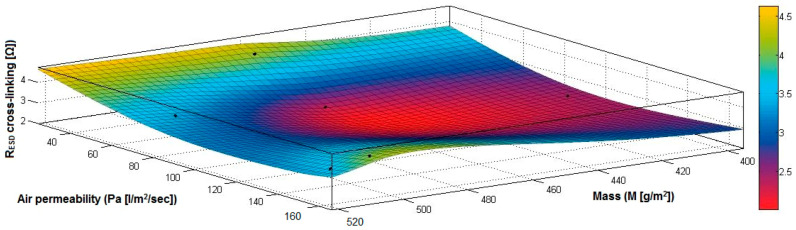
3D representation R_ESD after crosslinking_ = f(M, Pa) samples preliminarily treated in RF plasma O_2_ using RF_2_ generator and power 50 W.

**Figure 43:**
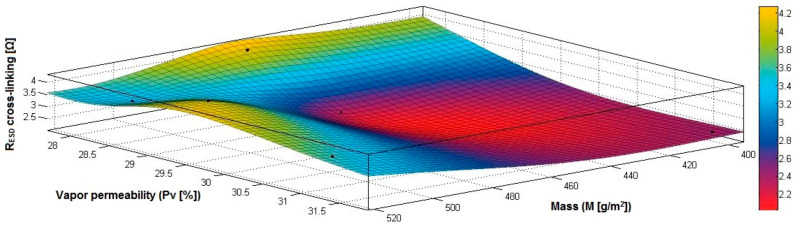
3D representation R_ESD after crosslinking_ = f(M, Pv) samples preliminarily treated in RF plasma O_2_ using RF_2_ generator and power 50 W.

In the case of the fabrics treated in RF plasma using RF_2_ and power 50 W, Figure 38, Figure 39, Figure 40, Figure 41, Figure 42 and Figure 43 present the 3D representation of the surface resistance (R_ESD after crosslinking_), after crosslinking, depending on the independent variables (mass and thickness (Figure 38), air permeability and thickness (Figure 39), vapor permeability and thickness (Figure 40), air permeability and vapor permeability (Figure 41), mass and vapor permeability (Figure 42) and mass and air permeability (Figure 43) to observe the variation of the R_ESD after crosslinking_ in correlation with the independent variables (mass, thickness, vapor permeability and air permeability).

In the case of the samples preliminarily treated using RF plasma O_2_ with RF_2_ generator and power 50 W, the correlation coefficients have been calculated (14)–(17):

(14) $$r_{R_{ESD},\;M}=\left|\begin{array}{cc} 1.0000 & 0.6297\\ 0.6297 & 1.0000 \end{array}\right|\Leftrightarrow r12_{R_{ESD},M}=r21_{R_{ESD},M}=0.6297$$

(15) $$r_{R_{ESD},\;Pa}=\left|\begin{array}{cc} 1.0000 & 0.2382\\ 0.2382 & 1.0000 \end{array}\right|\Leftrightarrow r12_{R_{ESD},Pa}=r21_{R_{ESD},Pa}=0.2382$$

(16) $$r_{R_{ESD},\;\delta }=\left|\begin{array}{cc} 1.0000 & 0.5099\\ 0.5099 & 1.0000 \end{array}\right|\Leftrightarrow r12_{R_{ESD},\delta }=r21_{R_{ESD},\delta }=0.5099$$

(17) $$r_{R_{ESD},\;Pv}=\left|\begin{array}{cc} 1.0000 & -0.4407\\ -0.4407 & 1.0000 \end{array}\right|\Leftrightarrow r12_{R_{ESD},Pv}=r21_{R_{ESD},Pv}=-0.4407$$

Analyzing the correlation coefficients between R_ESD after crosslinking_ and M, Pa, Pv and δ ($r_{R_{ESD},\;M}=0.6297\;\left(14\right)$, $r_{R_{ESD},\;Pa}=0.2382\;\left(15\right)$ and $r_{R_{ESD},\;\delta }=0.5099\;\left(16\right)),$ we can observe the coefficients are positive, and there is a direct correlation between variables, which means that an increase in the surface resistance can be generated by increasing the M, Pa or δ. In addition, the correlation coefficient $r_{R_{ESD},\;Pv}$ (17) is negative, indicating an inverse correlation and inverse proportionality report between R_ESD_ and Pv.

Figure 38 presents the 3D representation of the multiple correlations between R_ESD after crosslinking_ and (M, δ) and the black dots represent the fitting points for R_ESD after crosslinking_ depending on mass (M) and thickness (δ).

Figure 39 presents the 3D representation of the multiple correlations between R_ESD after crosslinking_ and (Pa, δ) and the black dots represent the fitting points for R_ESD after crosslinking_ depending on air permeability (Pa) and thickness (δ).

Figure 40 presents the 3D representation of the multiple correlations between R_ESD after crosslinking_ and (Pv, δ) and the black dots represent the fitting points for R_ESD after crosslinking_ depending on vapor permeability (Pv) and thickness (δ).

Figure 41 presents the 3D representation of the multiple correlations between R_ESD after crosslinking_ and (Pa, Pv) and the black dots represent the fitting points for R_ESD after crosslinking_ depending on air permeability (Pa) and vapor permeability (Pv).

Figure 42 presents the 3D representation of the multiple correlations between R_ESD after crosslinking_ and (M, Pa) and the black dots represent the fitting points for R_ESD after crosslinking_ depending on mass (M) and air permeability (Pa).

Figure 43 presents the 3D representation of the multiple correlations between R_ESD after crosslinking_ and (M, Pv) and the black dots represent the fitting points for R_ESD after crosslinking_ depending on mass (M) and vapor permeability (Pv).

## 5. Conclusions

In conclusion, the samples treated in RF plasma O_2_ using RF_1_ generator at 13.56 MHz frequency and power 200 W and using RF_2_ generator at 40 kHz frequency and 100 W power present an excellent hydrophilic character only in 3 min compared to samples treated in RF plasma O_2_ using the RF_2_ generator at 40 kHz frequency and power 50 W.

Moreover, in the case of samples functionalized in plasma and coated with PVA paste-based Ni particles, it was observed that the samples become conductive after drying at room temperature (18–20 °C) for 20–24 h.

In addition, in the samples hydrophobized with NUVA TTC and treated with polymeric paste-based PVA matrix and Cu microparticles, after crosslinking, the surface resistance was increased ten times, from 10^9^ Ω to 10^10^ Ω, and the resurface resistance value is a specific value for antistatic fabrics.

Analyzing the correlation coefficients, we can conclude that the correlation coefficients between R_ESD after crosslinking_ and M, δ and Pv are negative in the case of the samples pretreated in RF plasma O_2_ using RF_1_ generator and 200 W power, and this indicates a strong inverse correlation and an inverse proportionality relationship between these variables. However, the correlation coefficient between R_ESD after crosslinking_ and Pa is positive, and this shows a direct correlation and direct proportionality between these variables and suggests that increasing the Pa (due to the textile structure and air gaps in the fabric because the air is an excellent electrical insulator) will generate the increasing of the R_ESD after crosslinking_.

The fabrics that are treated in RF plasma O_2_ using RF_1_ generator and power 200 W and RF_2_ generator and 100 W power, followed by coating with a thin film based on PVA matrix, organic solvents and Ni microparticles present a good conductivity, reduced surface resistance and can be used for sensors development.

The development of conductive textiles for flexible electrodes using clean technology such as RF plasma oxygen leads to decreasing the wastewater, chemicals, carbon footprint and pretreatment time.

## Figures and Tables

**Table 1:** RF O_2_ plasma process parameters and electrical characterization of the samples after coating with polymeric pastes based on PVA and Ni microparticles.

No	Rs [Ω]	R_ESD_ Free Drying	R_ESD_ Cross-Linking	RF_1_ Plasma O_2_ Using MHz Generator				Physical-Mechanical Characteristics			
				Power [W]	Gas Flow [sccm]	Time [min]	Presssure [mTorr]	* M [g/m^2^]	** δ [mm]	*** Pv [%]	**** Pa [L/m^2^/s]
1	10^3^	11.7	0.25	200	200	3	100.4	503.6	1.36	31.88	148.8
2	10^3^	12.8	0.61	200	200	3	100.4	492.8	1.296	30.73	171.8
3	10^3^	10.6	0.4	200	200	3	100.4	398.4	1.014	32.26	92.82
4	10^3^	6	0.6	200	200	3	100.4	453.2	1.074	30.15	67.84
5	10^3^	6.8	0.04	200	200	3	100.4	470.8	1.366	31.11	74.84
6	10^3^	3.3	0.02	200	200	3	100.4	476.8	1.322	30.34	90.4
7	10^3^	7.8	0.07	200	200	3	100.4	454.8	1.12	31.11	46.44
8	10^3^	4.5	7.69	200	200	3	100.4	431.6	0.814	26.69	130
**RF Plasma O_2_ using RF_2_ kHz generator**											
9	10^3^	-	0.16	100	200	3	84.8	522	1.39	32.26	161.2
10	10^3^	-	2.69	100	200	3	84.8	510.4	1.36	32.67	184.6
11	10^3^	-	0.01	100	200	3	84.8	410	1.01	35.74	88.12
12	10^3^	-	0.01	100	200	3	84.8	477.6	1.09	34.72	64.12
13	10^3^	-	0.17	100	200	3	84.8	512.4	1.36	32.67	67.18
14	10^3^	-	0.01	100	200	3	84.8	502.4	1.36	30.84	97.36
15	10^3^	-	0.04	100	200	3	84.8	473.2	1.15	33.08	47.92
16	10^3^	-	7.09	100	200	3	84.8	437.2	0.81	29	120.8
17	10^12^	-	3.53	50	200	3	93.1	516.4	1.39	31.2	157.2
18	10^12^	-	4.03	50	200	3	93.1	506.4	1.294	29.2	160.4
19	10^12^	-	2.39	50	200	3	93.1	401.2	1.002	31.7	90.8
20	10^12^	-	2.31	50	200	3	93.1	456.8	1.064	29	60.82
21	10^13^	-	3.37	50	200	3	93.1	508.8	1.412	28.3	70.1
22	10^12^	-	4.07	50	200	3	93.1	459.6	1.084	27.9	31.12

* M—mass[g/m^2^], ** δ—thickness [mm], *** Pv—Vapor permeability [%], **** Pa—Air permeability [l/m^2^/s].

**Table 2:** Physico-mechanical and electrical characterization of the experimental samples (raw fabrics) functionalized by coating with polymeric pastes based PVA—and metallic microparticles Cu, Ni and Ag.

No.	Nuva TCC	Ni	Cu	Ag	PVA	H_2_O	* Rs_1_ [Ω]	** Rs_2_ [Ω]	M [g/m^2^]	δ [mm]	Pa [l/m^2^/s]
A1			x		x	x	10^9^	10^12^	992.8	2.932	8.148
A2		x			x	x	10^3^	10^3^	950.4	3.9	10.148
A3				x	x	x	10^3^	10^3^	1020.4	3.248	3.248
A4	x		x		x	x	10^9^	10^10^	1125.2	4.106	90.3
A5	x	x			x	x	10^3^	10^3^	966.4	4.00	90.383
A6	x			x	x	x	10^8^	10^3^	1002.8	4.762	141

* Rs_1_ measured before crosslinking; ** Rs_2_ measured after crosslinking.

**Table 3:** Surface topography of the textiles functionalized in RF plasma O_2_ using RF_1_ generator.

Sample No.	After Hydrophilization in RF Plasma O_2_ Using RF_1_ Generator	Surface After Deposition of Thin-Film Based PVA Matrix and Nickel Microparticles
1	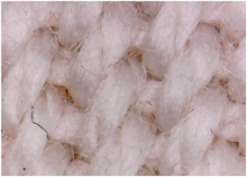	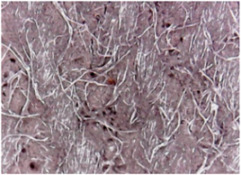
2	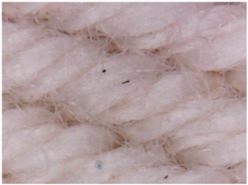	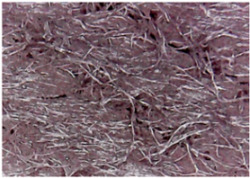
3	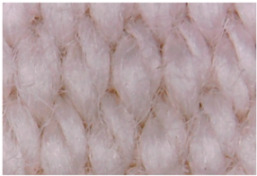	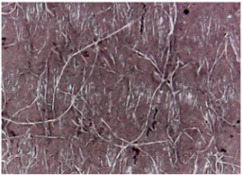
4	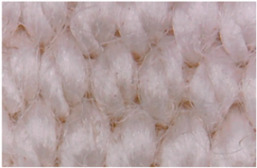	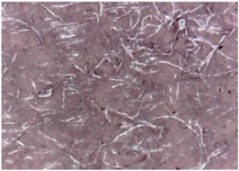
5	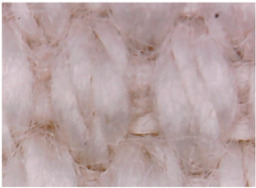	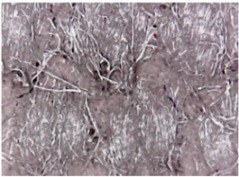
6	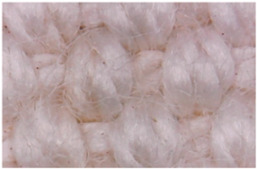	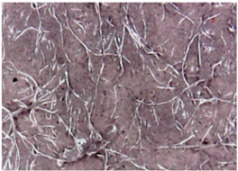

**Table 4:** Topography analysis of the samples obtained by classical technologies: boiling-bleaching (samples A1–A3) and alkaline boiling-bleaching followed by hydrophobization using NUVA TTC (samples A4–A6) and scraping of conductive pastes based on PVA matrix and Ni, Cu and Ag microparticles.

Microparticles	Hydrophil Fabric	Hydrophobic Fabric
Ni	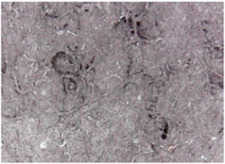	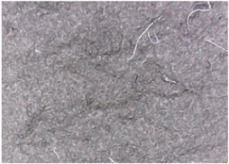
Cu	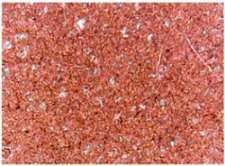	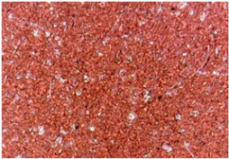
Ag	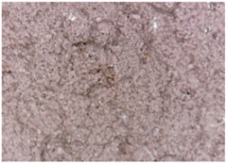	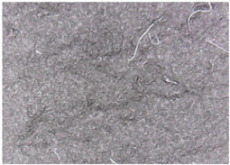

**Table 5:** Chemical composition (% wt).

Sample No.	C	O	Cl	K	Ni
S1_treated using RF2, 50 W_	38	53	3	6	-
S1_treated with Ni paste_	31	36	2	4	26
S2_treated using RF1, 200 W_	39	54	2	2	-
S2 _treated with Ni paste_	7	9	-	-	83
S3_treated using RF2, 100 W_	40	54	3	4	-
S3 _treated with Ni paste_	9	11	-	2	78

## Data Availability

Not applicable.

## References

[1] Höcker H. (2002). Plasma treatment of textile fibers. Pure Appl. Chem..

[2] Rauscher H., Perucca M., Buyle G. (2010). Plasma Technology for Hyperfunctional Surfaces: Food, Biomedical and Textile Applications.

[3] Takamatsu T., Hirai H., Sasaki R., Miyahara H., Okino A. (2012). Surface hydrophilization of polyimide films using atmospheric damage-free multigas plasma jet source. IEEE Trans. Plasma Sci..

[4] Kale K., Palaskar S. (2011). Atmospheric Pressure Glow Discharge of Helium-Oxygen Plasma Treatment on Polyester/Cotton Blended Fabric. Indian J. Fibre Text. Res..

[5] Kamel M.M., El Zawahry M.M., Helmy H., Eid M.A. (2011). Improvements in the dyeability of polyester fabrics by atmospheric pressure oxygen plasma treatment. J. Text. Inst..

[6] Mehmood T., Kaynak A., Dai X.J., Kouzani A., Magniez K., de Celis D.R., Hurren C.J., du Plessis J. (2014). Study of oxygen plasma pre-treatment of polyester fabric for improved polypyrrole adhesion. Mater. Chem. Phys..

[7] Wei Q., Wang Y., Yang Q., Yu L. (2007). Functionalization of Textile Materials by Plasma Enhanced Modification. J. Ind. Text..

[8] Vesel A., Mozetic M., Strnad S., Peršin Z., Stana-Kleinschek K., Hauptman N. (2009). Plasma modification of viscose textile. Vacuum.

[9] Maalek R., Holme I. (2003). The Effect of Plasma Treatment on Some Properties of Cotton. Iran. Polym. J..

[10] Wang Q., Fan X.R., Cui L., Wang P., Wu J., Chen J. (2009). Plasma-aided cotton bioscouring: Dielectric barrier discharge versus low-pressure oxygen plasma. Plasma Chem. Plasma Process..

[11] Jazbec K., Šala M., Mozetič M., Vesel A., Gorjanc M. (2015). Functionalization of Cellulose Fibres with Oxygen Plasma and ZnO Nanoparticles for Achieving UV Protective Properties. J. Nanomater..

[12] Wang C., Lv J., Ren Y., Zhou Q., Chen J., Zhi T., Lu Z., Gao D., Ma Z., Jin L. (2016). Cotton fabric with plasma pretreatment and ZnO/Carboxymethyl chitosan composite finishing for durable UV resistance and antibacterial property. Carbohydr. Polym..

[13] Lam Y.L., Kan C.W., Yuen C.W. (2011). Effect of oxygen plasma pretreatment and titanium dioxide overlay coating on flame retardant finished cotton fabrics. Bioresources.

[14] Mejía M., Marín J., Restrepo G., Pulgarín C., Mielczarski E., Arroyo Y., Lavanchy J.-C., Kiwi J. (2009). Self-cleaning modified TiO2–cotton pretreated by UVC-light (185 nm) and RF-plasma in vacuum and also under atmospheric pressure. Appl. Catal. B Environ..

[15] Shahidi S., Rashidi A., Ghoranneviss M., Anvari A., Rahimi M.K., Moghaddam M.B., Wiener J. (2010). Investigation of metal absorption and antibacterial activity on cotton fabric modified by low temperature plasma. Cellulose.

[16] Lam Y.L., Kan C.-W., Yuen C.W.M. (2013). A study of metal oxide on antimicrobial effect of plasma pre-treated cotton fabric. Fibers Polym..

[17] Hassabo A.G., El-Sayed E. (2021). Recent advances in the application of plasma in textile finishing (A Review). J. Text. Color. Polym. Sci..

[18] Malapit G.M., Baculi R.Q. (2021). Bactericidal efficiency of silver nanoparticles deposited on polyester fabric using atmospheric pressure plasma jet system. J. Text. Inst..

[19] Haji A. (2013). Eco-friendly dyeing and antibacterial treatment of cotton. Cellul. Chem. Technol..

[20] Vaideki K., Jayakumar S., Thilagavathi G., Rajendran R. (2007). A study on the antimicrobial efficacy of RF oxygen plasma and neem extract treated cotton fabrics. Appl. Surf. Sci..

[21] Katouah H., El-Metwaly N.M. (2021). Plasma treatment toward electrically conductive and superhydrophobic cotton fibers by in situ preparation of polypyrrole and silver nanoparticles. React. Funct. Polym..

[22] Jia Y., Xin B. (2021). Preparation and Characterization of Polypyrrole-coated Wool Fabric for High Electrical Conductivity. J. Phys. Conf. Ser..

[23] Hu S., Kremenakova D., Militký J., Periyasamy A.P. (2021). Copper-Coated Textiles for Viruses Dodging. Textiles and Their Use in Microbial Protection.

[24] Kogelschatz U. (2003). Dielectric-Barrier Discharges: Their History, Discharge Physics, and Industrial Applications. Plasma Chem. Plasma Process..

[25] Hossain M.M., Herrmann A.S., Hegemann D. (2006). Plasma Hydrophilization Effect on Different Textile Structures. Plasma Process. Polym..

[26] Haji A., Kan C.-W. (2021). Plasma Treatment for Sustainable Functionalization of Textiles.

[27] Gouveia I.C., Antunes L.C., Gomes A.P. (2011). Low-pressure plasma treatment for hydrophilization of poly(ethylene terephthalate) fabrics. J. Text. Inst..

[28] Chen F., Xu W., Huang S., Liu J., Song J., Liu X. (2016). Plasma Hydrophilization of Superhydrophobic Surface and Its Aging Behavior: The Effect of Micro/nanostructured Surface. Surf. Interface Anal..

[29] Bogaczyk M., Sretenović G.B., Wagner H.-E. (2013). Influence of the applied voltage shape on the barrier discharge operation modes in helium. Eur. Phys. J. D.

[30] Saifutdinov A.I., Saifutdinova A.A., Timerkaev B.A. (2018). Numerical Study of the Voltage Waveform Effect on the Spatio-temporal Characteristics of a Dielectric Barrier Microdischarge in Argon. Plasma Phys. Rep..

[31] Abdelaziz A.A., Ishijima T., Seto T. (2018). Humidity effects on surface dielectric barrier discharge for gaseous naphthalene decomposition. Phys. Plasmas.

[32] Mazharul I.K. Chemical Composition of the Cotton Fiber. textilelearner.net/chemical-composition-of-cotton-fiber.

[33] Pettigrew W.T. (2008). Potassium influences on yield and quality production for maize, wheat, soybean and cotton. Physiol. Plant..

[34] Hussain S., Ali H., Gardezi S.T.R. (2021). Soil applied potassium improves productivity and fiber quality of cotton cultivars grown on potassium deficient soils. PLoS ONE.

[35] Fontana J.E., Wang G., Sun R., Xue H., Li Q., Liu J., Davis K.E., Thornburg T.E., Zhang B., Zhang Z. (2020). Impact of potassium deficiency on cotton growth, development and potential microRNA-mediated mechanism. Plant Physiol. Biochem..

